# Experimental Data Based Machine Learning Classification Models with Predictive Ability to Select in Vitro Active Antiviral and Non-Toxic Essential Oils

**DOI:** 10.3390/molecules25102452

**Published:** 2020-05-25

**Authors:** Manuela Sabatino, Marco Fabiani, Mijat Božović, Stefania Garzoli, Lorenzo Antonini, Maria Elena Marcocci, Anna Teresa Palamara, Giovanna De Chiara, Rino Ragno

**Affiliations:** 1 Rome Center for Molecular Design, Department of Drug Chemistry and Technology, Sapienza University, P.le Aldo Moro 5, 00185 Rome, Italy; manuela.sabatino@uniroma1.it (M.S.); lorenzo.antonini@uniroma1.it (L.A.); 2 Department of Public Health and Infectious Diseases, Sapienza University of Rome, Laboratory affiliated to Istituto Pasteur Italia–Fondazione Cenci Bolognetti, 00161 Rome, Italy; marco.fabiani@uniroma1.it (M.F.); mariaelena.marcocci@uniroma1.it (M.E.M.); 3 University of Montenegro, Faculty of Science and Mathematics, Džordža Vašingtona bb, 81000 Podgorica, Montenegro; mijatboz@ucg.ac.me; 4 Department of Drug Chemistry and Technology, Sapienza University, P.le Aldo Moro 5, 00185 Rome, Italy; stefania.garzoli@uniroma1.it; 5 San Raffaele Pisana, IRCCS, Telematic University, 00166 Rome, Italy; 6 Institute of Translational Pharmacology, National Research Council, 00133 Rome, Italy; giovanna.dechiara@ift.cnr.it

**Keywords:** machine learning, classification modeling, essential oil, herpes simplex virus, PLS-DA, quantitative composition-activity relationships, QCAR, multidisciplinary application

## Abstract

In the last decade essential oils have attracted scientists with a constant increase rate of more than 7% as witnessed by almost 5000 articles. Among the prominent studies essential oils are investigated as antibacterial agents alone or in combination with known drugs. Minor studies involved essential oil inspection as potential anticancer and antiviral natural remedies. In line with the authors previous reports the investigation of an in-house library of extracted essential oils as a potential blocker of HSV-1 infection is reported herein. A subset of essential oils was experimentally tested in an in vitro model of HSV-1 infection and the determined IC_50_s and CC_50_s values were used in conjunction with the results obtained by gas-chromatography/mass spectrometry chemical analysis to derive machine learning based classification models trained with the partial least square discriminant analysis algorithm. The internally validated models were thus applied on untested essential oils to assess their effective predictive ability in selecting both active and low toxic samples. Five essential oils were selected among a list of 52 and readily assayed for IC_50_ and CC_50_ determination. Interestingly, four out of the five selected samples, compared with the potencies of the training set, returned to be highly active and endowed with low toxicity. In particular, sample CJM1 from *Calaminta nepeta* was the most potent tested essential oil with the highest selectivity index (IC_50_ = 0.063 mg/mL, SI > 47.5). In conclusion, it was herein demonstrated how multidisciplinary applications involving machine learning could represent a valuable tool in predicting the bioactivity of complex mixtures and in the near future to enable the design of blended essential oil possibly endowed with higher potency and lower toxicity.

## 1. Introduction

Essential oils (EOs) are natural complex aromatic-smelling mixture [1], deriving from plants’ secondary metabolism and containing predominately monoterpenes, sesquiterpenes and their oxygenated derivatives. EOs are known to be biosynthesized in flowers, leaves, fruits and roots [2] and are industrially produced mainly by hydro- [3] or steam-distillation [4].

**Figure 1:**
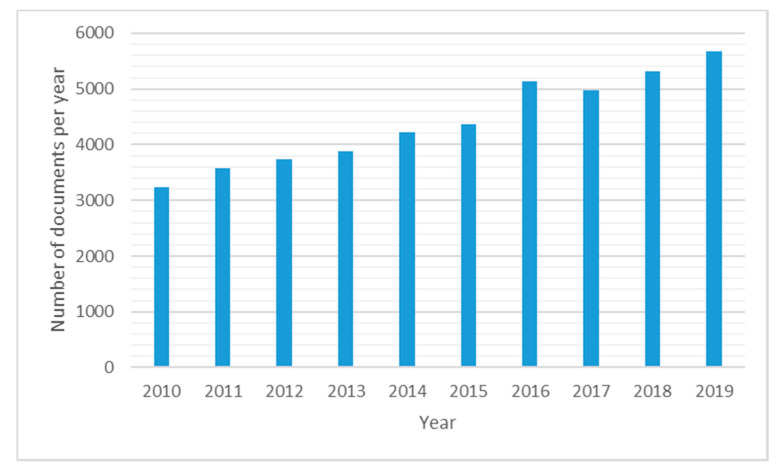
Trend of number of scientific articles on essential oils. Source scopus.com by using ‘essential’ and ‘oil’ as keywords (accessed in February 2020).

Interests in the use of EOs in several fields is continuously increasing due to their biological properties such as antioxidant or antimicrobial activities and many others [5,6,7,8]. Due to the EOs’ chemical composition complexity, recently challenges have been undertaken to discern the synergistic and anti-synergistic roles of each single constituent and how they could influence the pharmacological activities [9,10,11]. This categorization is even more complicated due to the ‘chemotype’ concept, in which the same plant could produce different EOs’ chemical composition profiles and therefore different biological properties [12,13]. Holy basil, thyme, lavender and peppermint are examples of plants with several chemotypes [14]. Despite this hurdle, an effort to characterize EOs is currently undergoing in medical and pharmaceutical fields, with the goal to obtain a clearer indication for their uses in traditional medicine, chemical or pharmaceutical as witnessed by almost 5000 articles in the last decade with an average positive increment of more than 7% per year (Figure 1).

The emergence of novel drug-resistant microorganisms motivates the continuous search for new therapeutic agents also in the natural world [15]. Among these, EOs are also continuously under investigation as potential new antiviral agents. EOs derived from *Malaleuca alternifolia*, *Mentha piperita* and *Thymus vulgaris* as well as isolated essential oil components, were reported to show antiviral properties, specifically against enveloped viruses [9]. In 2014 Civitelli et al. [16] explored *Mentha suaveolens* EO (MSEO) effectiveness against herpes simplex virus type-1 (HSV-1) replication in an in vitro model of infection. MSEO and its main component, piperitenone oxide, were found to reduce HSV-1 replication with IC_50_s of 0.0051 mg/mL and 0.0014 mg/mL, respectively. Very recently, Toujani et al. [17] demonstrated the antiviral properties of *Thymus capitatus* against HSV-1 and herpes simplex virus type-2 (HSV-2), by testing three different phytopreparations (aqueous extract (AE), ethanolic extract (EE) and EOs). *Thymus capitatus* phytopreparations AE, EE and EO were analyzed by a gas chromatography/mass spectrometry (GC/MS) technique [10,15,18,19], identifying β-sitosterol, cinnamaldehyde and carvacrol as the major chemical components. These three molecules were thus tested as pure compounds for their ability to inhibit the HSV-2 replication showing an EC_50_ of 0.0027, 0.0397 and 0.0519 mg/mL, respectively [17].

Multidisciplinary applications have been reported to successfully confirm the antiviral properties of some medicinal plants extracts, including EOs as recently reported by Tariq et al. [20]. In this context, the herein reported study was aimed at investigating the potential anti-HSV-1 activity on a series of EOs to improve the knowledge about the antiviral effects of natural chemical mixtures. Hence, a series of EOs derived from three different plants, *Calamintha nepeta* (CN) [18], *Foeniculum vulgare* (FV) [19] and *Ridolfia segetum* (RS) [21], were considered. These EOs, extracted using the protocol by Božović et al. [18] and chemically characterized by GC/MS were herein tested in an in vitro model of HSV-1 infection. Next, by means of principal component analysis (PCA) [22] and partial least squares discriminant analysis (PLS-DA) [23], quantitative composition-activity relationships (QCAR) models were developed and validated for their abilities in prediction to select further untested EOs for improved antiviral and cytotoxic profile or possibly design blended EOs [24,25].

## 2. Results and Discussion

### 2.1. EOs’ Cytotoxic and Antiviral Effects

**Figure 2:**
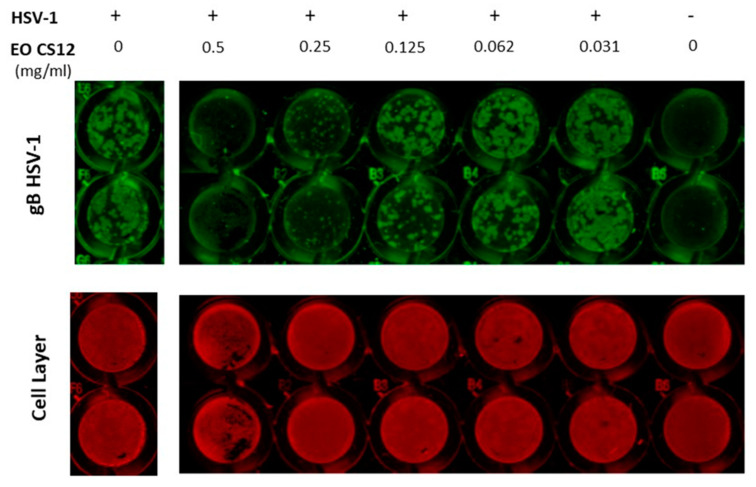
In cell western (ICW) analysis of anti-HSV-1 effects of EO (68, CS12). 0.1 m.o.i. HSV-1-infected Vero cells were treated for 24 h with serial dilutions of EO 68 ranging from 0.0312 to 0.5 mg/mL. Untreated HSV-1-infected cells (0 mg/mL EO) were used as comparative control. Fixed cells were incubated with anti-gB primary antibody and then with IRDye 800 CW secondary antibody (green fluorescence) and CellTag 700 Stain (red fluorescence). HSV-1 is a mock infected cell (used as staining control).

First, to check for cytotoxicity, Vero cells were incubated with different EO concentrations (0.001–0.5 mg/mL) for 24 h and cell proliferation was measured by means of MTT assay (Figure 2). Then the antiviral effect was evaluated in Vero cells infected with 0.1 m.o.i. of HSV-1 and exposed soon after the virus-adsorption period (1 h) to various concentrations of each EO in a range of 0.0312–0.5 mg/mL for 24 h post-infection (p.i.; a representative ICW analysis is shown in Figure 1, results in Table 1). With the only exception of samples 9 (FO24) and 35 (R6), all tested EOs displayed CC_50_ values higher than IC_50_s, thus indicating that their effect on viral replication was not affected by the cytotoxicities (Table 2). In particular, EOs from CN displayed the highest antiviral potencies (IC_50_ range = 0.12–0.44 mg/mL, average = 0.22 mg/mL), the lowest cytotoxicity (CC_50_ range = 1.10–4.71 mg/mL, average = 2.65 mg/mL) and the most favorable selectivity indexes (SI range = 2.50–34.57, average = 13.87). Intermediate favorable profile was displayed by FVEOs samples that had average values of IC_50_, CC_50_ and SI of 0.356, 1.24 and 4.7, respectively. Regarding the four RSEO samples, they displayed the worst profile with good average IC_50_, but associated to high cytotoxicity and thus low SI values.

### 2.2. Machine Learning Modeling

#### 2.2.1. Unsupervised Data Analysis

**Figure 3:**
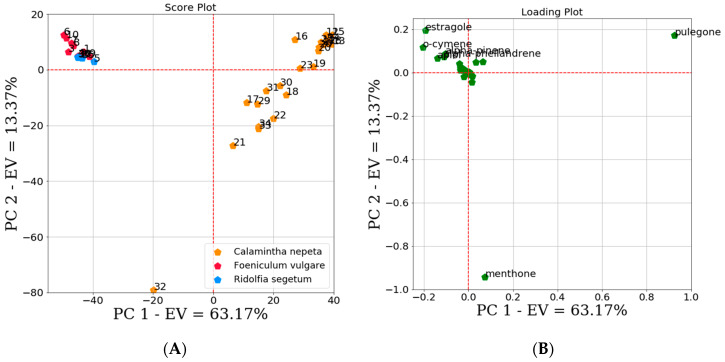
Scores (**A**) and loadings (**B**) plots of the first two principal components (PCs). EV indicates the explained variance. Score plot (**A**) showed the presence of two clusters, a first cluster indicated the *Calamintha Nepeta* (CNEO) samples, the second one *Foeniculum vulgare* (FVEO) and *Ridolfia Segetum* (RSEO) samples. In the plot sample 32 seems to be the outlier. The first two PCs in loading plot (**B**) highlight that estragole, o-cymene, alpha-pinene, alpha-phellandrene and pulegone could be the most important chemical constituents among all the tested EOs.

PCA was used as an unsupervised technique to analyze and compare the 38 selected EOs (Table 2). A cumulative explained variance of about 76.54% was described by the first two PCs. In particular, 63.17% of data variability was contained in the first PC, while 13.37% in the second PC. A cumulative explained variance of 95% was obtained extracting the third and fourth PCs (PC3 = 11.41%, PC4 = 7.04%). As most of the variance was contained in the first two PCs inspection was focused at the respective score plot whose analysis revealed the presence of two distinct clusters (Figure 3A). FVEOs and RSEOs were grouped in a first most populated cluster, whereas the CNEOs constituted a second one. The two clusters clearly indicated differences in the EOs chemical compositions and at the same time also some resemblances among RSEOs and FVEOs samples. Analysis of PCA loading plots (Figure 3B) revealed estragole, o-cymene, α-pinene and α-phellandrene as the chemical constituents mainly characterizing RSEOs and FVEOs cluster, while pulegone was mainly associated to the CNEOs samples cluster. A further important chemical component emphasized by the PCA loading plot (Figure 3B) was menthone, mainly associated to sample 32 (COM1) that seem to be of peculiar composition so that this sample in the score plot is localized in a zone not comprised in neither above clusters.

#### 2.2.2. Supervised Classification Modeling

Optimal cut-off values to divide the dependent data (IC_50_ and CC_50_), into active (A) and non-active (NA), toxic (T) and non-toxic (NT) classes were established starting from the corresponding median values (0.20 for the IC_50_ and 2.06 for the CC_50_), which were systematically modified applying an increase or decrease of 5% to inspect for different cut-off boundaries. Cut-off values were inspected while running leave one out cross validation (LOO-CV) while monitoring the explained variance (EV), the fitting-non-error-rate (FNER), the cross-validation-non-error-rate (CVNER) and accuracy (ACC; Table 1).

For IC_50_ values, the best PLS-DA classification model (IC_50_-PLS-DA) was obtained with a cut-off of 0.15, while for CC_50_s (CC_50_-PLS-DA) the optimal cut-off was found to be 2.06 (Table 3).

**Figure 4:**
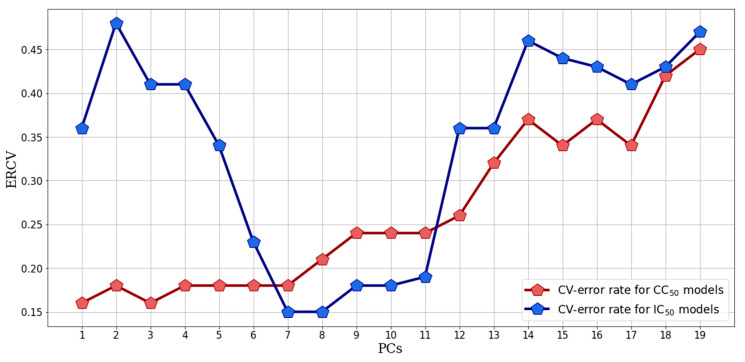
Cross validation error rate (CVER) as a function of the increasing number of the number of latent variables included in the PLS-DA preliminary model. Blue line represents the CVER trend for the IC_50_-PLS-DA, the red one the ERCV trend for the CC_50_-PLS-DA. The red and blue pentagons represent the CVER values obtained for the model built with the number of PCs indicated on the x-axes.

Contiguous blocks LOO-CV with 19 PCs was applied to a preliminary model in order to select the optimal number of latent variables to be used for either IC_50_-PLS-DA or CC_50_-PLS-DA datasets. Focusing on IC_50_-PLS-DA, the analysis of the cross validation error rate (CVER) as a function of the increasing number of PCs showed a minimum explained variance difference between 7 and 8 PCs, revealing the first one as the best PC to be used in the final model (Figure 4). A similar analysis was performed for CC_50_-PLS-DA, identifying the lowest CVER value both in 1 and the 3 PCs. To guarantee a major explained variance 3 PC was set (Figure 4).

**Figure 5:**
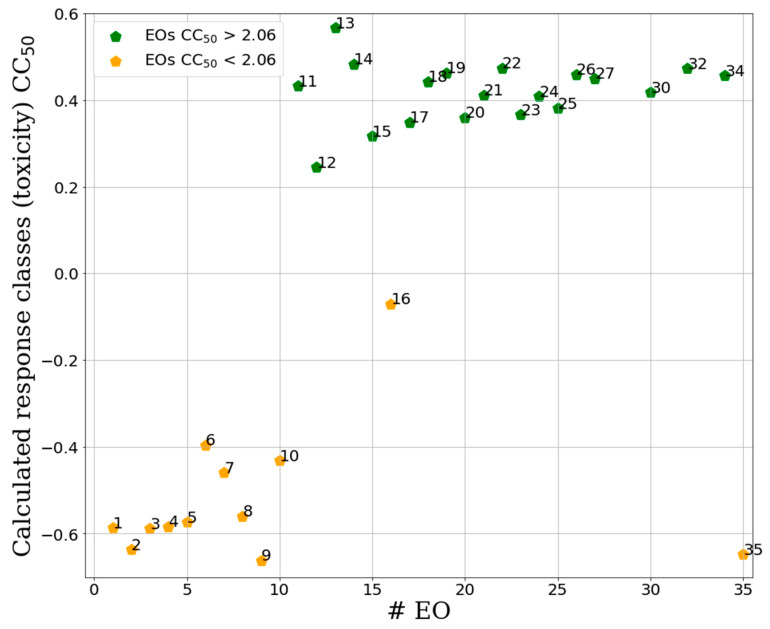
Separation between active and inactive samples for the binary CC_50_-PLS-DA classification model. Green samples are the non-toxic class, orange the toxic. On the y-axis are reported the calculated response for the toxic classis calculated by the PLS-DA technique (X score × Y weights) for each EO [24] (Table 2). Samples at the top of the plot were assigned to be a non-toxic class while those at the bottom were classified as toxic.

**Figure 6:**
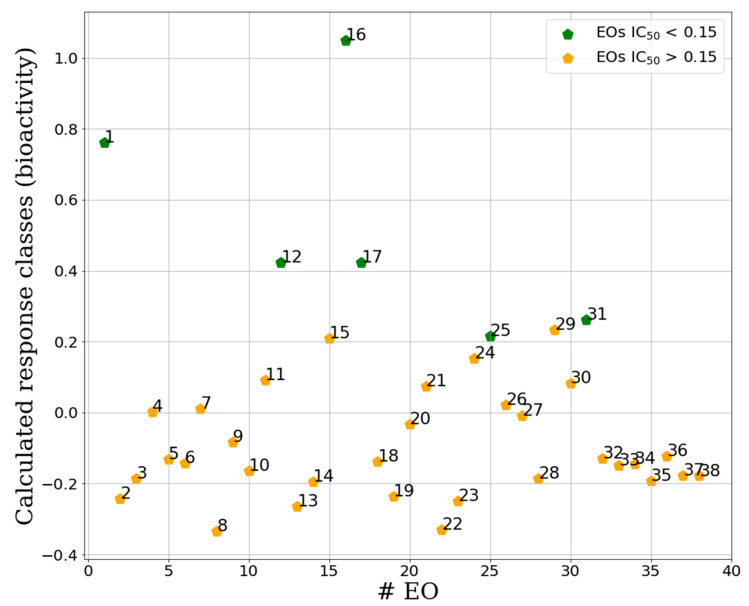
Separation between active and inactive samples for the binary IC_50_-PLS-DA classification model. Green samples are the active class, orange the inactive. On the y-axis are reported the calculated response for the activity class calculated by the PLS-DA technique (X score × Y weights) for each EO (x-axis) [24] (Table 2). Samples at the top of the plot were assigned to active class while those at the bottom were classified as inactive. Calculated response classis values between 1 and 0 indicate active samples, while values lower than 0 indicate samples belonging to the inactive class.

The IC_50_-PLS-DA model obtained with 7 PCs was characterized by FNER, CVNER and ACC values of 0.97, 0.85 and 0.87, respectively, indicating a good and reliable classification model. The graphical inspection of the samples class recalculation plot of IC_50_-PLS-DA model revealed quite complete separation between the two defined EO classes (actives and non-actives, Figure 5). In particular, the IC_50_-PLS-DA classification model recognized all active samples (**1**, **12**, **16**, **17**, **25** and **31**) as actives (green points in Figure 6), nevertheless two nonactive samples, namely **15** and **29,** were erroneously recalculated as actives. A high quality and robust CC_50_-PLS-DA model was instead obtained, characterized by 1.00, 0.97 and 0.97 values of FNER, CVNER and ACC, respectively, being able to divide the samples in two definite clusters (toxic and non-toxic) with 3 PCs. The CC_50_-PLS-DA classification model, obtained with a cut-off value of 2.06 was able to separate the more toxic samples (**1**–**4**, **6**, **9**–**11**, **13**–**20**, **22** and **23**) from the less toxic ones (**21**, **25**, **27**–**38**) (Figure 5).

**Figure 7:**
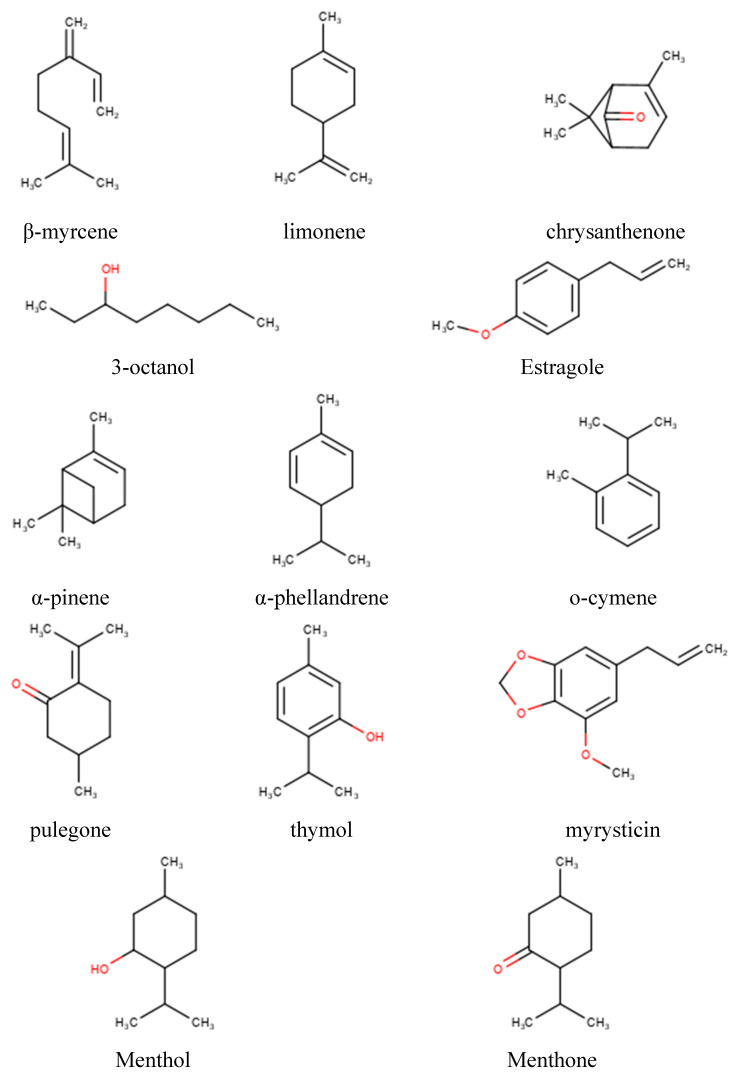
Chemical structures of the most important chemical components.

**Figure 8:**
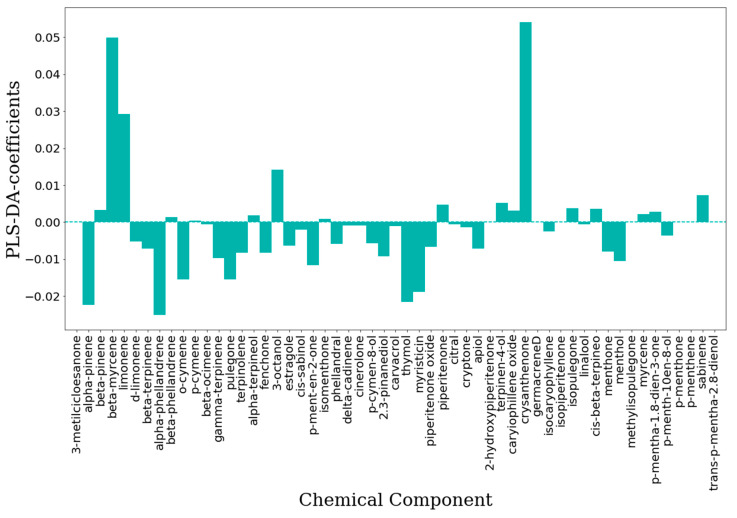
Features importance plot obtained from the IC_50_-PLS-DA classification model. On the ordinates are reported the PLS-DA coefficients while in the abscises are listed the chemical components.

The IC_50_-PLS-DA and CC_50_-PLS-DA classification models were also inspected by means of the features importance plot, in which is summarized the contribution of each chemical constituent to the biological and toxicology properties, respectively. For the antiviral effects, in the IC_50_-PLS-DA model β-myrcene, limonene, 3-octanol and crysanthenone (Figure 7) were characterized by positive PLS coefficients (Figure 8) that could represent those components able to differentiate EOs into active or inactive. On the other hand, by inspecting the negative PLS-DA coefficients (Figure 8), these indicated those compounds likely determining the decreased biological activity. Among those were α-pinene, α-phellandrene, o-cymene, pulegone, thymol and myristicin (Figure 7). Interestingly, some of these were pointed by the unsupervised analysis (PCA) as the chemical constituents characterizing the RSEOs and FVEOs cluster (Figure 3). The only exception was pulegone, associated to negative regression coefficient, but characterizing the cluster of CNEO samples labeled as active and non-toxic EOs. 3-metilcicloesanone, germacrene D, isopiperitenone, methylisopulegone, p-menthone, p-menthene and trans-p-mentha-2,8-dienol had zero values for the PLS-DA coefficient (Figure 7), these molecules likely to be neutral for the biological activity.

**Figure 9:**
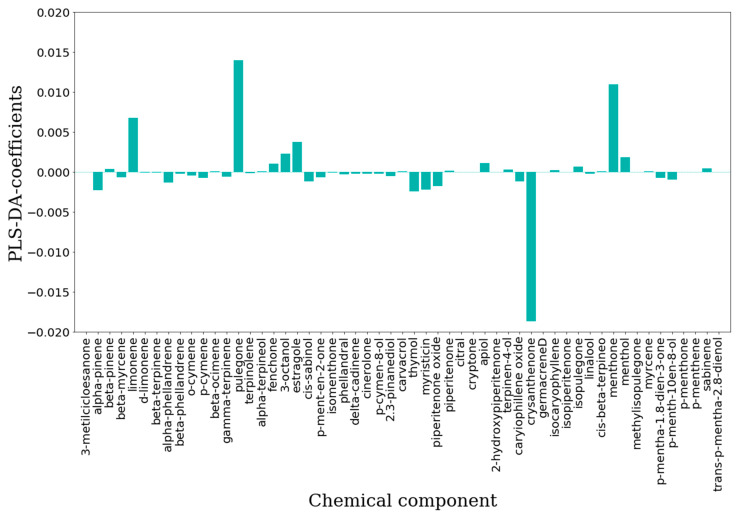
Features importance plot obtained from the CC_50_-PLS-DA- classification. On the y-axis are reported the PLS-DA coefficients while in the x-axis are listed the chemical components.

A similar analysis was carried out for CC_50_-PLS-DA classification model (Figure 9) positive coefficients were assigned to menthol, menthone, estragole, 3-octanol, pulegone and limonene (Figure 7) indicating that these compounds could be associated to an EOs with low toxicity profile.

Among chemical components characterized by negative PLS-DA coefficients, only chrysanthenone (Figure 7; Figure 9) displayed a highly negative coefficients indicating that it mainly associated to toxicity, nevertheless chrysanthenone displayed a positive coefficient in the IC_50_-PLS-DA model.

#### 2.2.3. PLS-DA Classification Models Predictive Abilities

**Figure 10:**
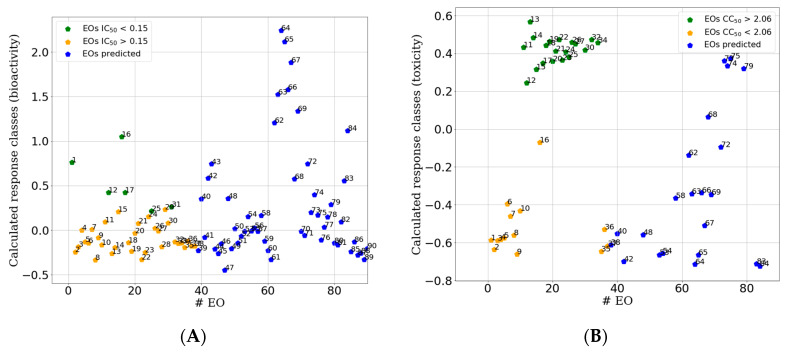
Sample plots for external test set (blue points) predicted by the IC_50_-PLS-DA classification (**A**) and by the CC_50_-PLS-DA classification. On the y-axis are reported the calculated response bioactivity classes (**A**) and toxicity class (**B**) by the PLS-DA model (X score × Y weights) [24] for all predicted EOs (test set, Table 2). For comparison in the plot are also reported the points relative to the training set (green and orange points, Figure 5).

As reported, any quantitative model should be assessed for its effective usability [26]. Herein the QCAR classification models were tested for their ability to classify the 52 excluded EOs used as an external test set. The two models were applied in a sequential way, as the first filter, the application of the above described IC_50_-PLS-DA classification model, predicted 21 out of 52 samples as potentially active against HSV-1 (**40**, **42**, **43**, **54**, **58**, **63**–**69**, **72**, **74**, **75**, **78**, **79**, **83** and **84** of Table 4, Figure 10A). Then, as a second filter, the CC_50_-PLS-DA classification model was applied on the 21 predicted active EOs and predicted only five of them as potentially endowed of low cytotoxicity (**68**, **73**–**75** and **79**; Figure 10B). Promptly the five EOs samples **68**, **73**–**75** and **79** were tested both for their ability to inhibit HSV-1 and for their cytotoxicity. Sample **68** was selected as proof of concept as it was predicted to be toxic. Surprisingly, the experimental data confirmed the predictions, revealing four out of five samples (80%) to be endowed of high anti-HSV-1 potency and low cytotoxicity. Indeed, two of the newly tested EOs (**73** and **75**) displayed IC_50_ with even greater potencies, being **73** the most potent (IC_50_ = 0.0632 mg/mL) and with increased selectivity index (SI = 47.5). In agreement with the prediction, sample **68** was indeed found with modest anti-HSV-1 potency, quite toxic and low SI index (Table 5).

## 3. Material and Methods

### 3.1. Plants Materials and EOs Extraction

EOs extracted from *Calamintha Nepeta* (CNEO), *Foeniculum vulgare* (FVEO) and *Ridolfia Segetum* (RSEO) plants were used in this study. In particular, 38 different EOs were selected from an in-house list of 90 EOs [10] on the basis of their chemical composition to cover as much as possible the chemical variability and reducing the experimental part [5,6]. As previously reported [10,27], aerial parts of the three plants were collected during the summer and early autumn periods of the year 2015, in a wild area around Tarquinia city (Province of Viterbo, Italy). As previously described [27], CNEOs were obtained directly from fresh plant material, while for FVEOs and RSEOs were used air-dried in a shady place for 20 days. EOs were extracted by steam distillation using a 62 L distillatory apparatus (Albrigi Luigi E0131, Verona, Italy), following the protocol previously reported [28]. To prevent degradation EOs’ were kept frozen at –30 °C until their usage and routinely checked for they stability.

EOs were dissolved in ethanol and further diluted in medium for cell culture experiments, always resulting in an ethanol concentration below 1%, which has no effect on cells and viruses [29].

### 3.2. GC/MS Analysis

The gas chromatographic/mass spectrometric (GC/MS) EOs analysis protocol was previously reported [15,27].

### 3.3. Cell Culture, Virus Production

African green monkey kidney ATCC CCL-81 Vero cells were grown in Roswell Park Memorial Institute (RPMI) 1640 medium (Gibco, Invitrogen Corporation, CA) supplemented with 10% heat-inactivated fetal bovine serum (FBS, Gibco, Invitrogen Corporation, CA), 1% glutamine, 50 U per mL penicillin and 50 μg/mL streptomycin (Sigma–Aldrich, MO, USA). The cells were maintained at 37 °C in humidified air containing 5% CO_2_. Viability of cells was estimated by Trypan blue (0.02% final concentration) exclusion assay (Invitrogen Corporation). For virus production monolayers of Vero cells in 75 cm^2^ tissue culture flasks were infected with HSV-1 strain F at a multiplicity of infection (m.o.i.) of 0.01. After 48 h at 37 °C, infected cells were harvested with 3 freeze-and-thaw cycles, cellular debris were removed with low-speed centrifugation and the virus titer was measured by the standard plaque assay [30]. Similarly, mock solution consists of the supernatant of mock-infected Vero cells. The titer of the virus preparation was 5 × 108 plaque forming units (pfu)/mL. The virus was stored at −70 °C until used.

### 3.4. Cellular Toxicity

Cellular toxicity of EOs was tested in vitro, as previously reported [31,32]. Monolayers of Vero cells were incubated with each of the 38 EOs at concentrations from 0.001 to 0.5 mg/mL in RPMI 1640 for 24 h and the medium added with 50 μL of a 1 mg/mL solution of MTT (3-(4,5-dimethylthiazol-2-yl)-2,5-diphenyl tetrazolium bromide, Sigma–Aldrich (St. Louis, MO)) in RPMI without phenol red (Sigma–Aldrich). Cells were incubated at 37 °C for 3 h, and 100 μL of acid-isopropanol (0.1 N HCl in isopropanol) was added to each well. After a slightly mixing by pipetting to ensure that all MTT crystals were dissolved, the plates were read using an automatic plate reader with a 570 nm test wavelength and a 690 nm reference wavelength. The drug concentration required for reducing the cell viability by 50% (CC_50_) was assessed. Wells containing medium with ethanol at the same concentration as in the samples were also included on each plate as controls.

### 3.5. In Cell Western (ICW) Technique for Antiviral Activity

The antiviral activity of EOs was evaluated using the in cell western (ICW) technique [33]. Briefly, Vero cells were seeded in 96 well-plates and after 24 h were HSV-1 infected at 0.1 m.o.i. and after 1 h adsorption at 37 °C, the plates were washed with phosphate buffered saline (PBS) and the medium replaced with 2% FBS RPMI containing 1% glutamine, 50 U per mL penicillin, and 50 µg/mL streptomycin in the presence of EOs at different serial concentrations (0.50, 0.25, 0.125, 0.0625 and 0.0312 mg/mL). HSV-1-infected cells cultured in the presence of EO vehicle were used as comparative control. Twenty-four hour later, cells were fixed with 4% paraformaldehyde in PBS for 15 min at room temperature (r.t.), and then were permeabilized in 0.1% triton X-100 PBS for 5 min at r.t. Cells were then incubated with Odyssey Blocking Buffer for 1 h at r.t., and then stained 1 h with a primary antibody raised against glycoprotein B (gB; sc-56987, Santa Cruz, 1:1000 dilution in Odyssey Blocking buffer), a late HSV-1 protein, then washed three times with PBS containing 0.1% Tween-20 and incubated with the secondary antibody IRDye 800 CW Goat Anti Mouse (926-32210 LI-COR Biosciences, 1:1000 dilution in Odyssey Blocking buffer; green fluorescence). Finally, cells were stained for 1 h with Cell-Tag 700 (926-41090, LI-COR Biosciences, 1:500) a fluorescent dye that stains cells and allows one to detect the cell layer (red fluorescence) in order to normalize viral protein fluorescence intensity to the cells number. After four washes with PBS containing 0.1% Tween-20, the plate was scanned on the Odyssey Infrared Imager, and the integrated intensity value of each well read by LI-COR Image Studio Software developed for Odyssey analysis. Mock-infected cells were used as controls, and their intensity used as a background. Normalized fluorescence intensity resulting from each staining was used to evaluate viral replication. Wells containing medium with ethanol at the same concentration as in the samples were also included on each plate as controls.

### 3.6. Biological Data Analysis

Data analysis for antiviral activity of EOs by ICW was evaluated using a method developed by exploiting a Java-based image processing program (IMAGE-J) that allows one to identify the surface area occupied by fluorescence in each well and then to calculate the ‘area percentage’, i.e., the percentage of well area covered by fluorescence [34]. The resulting values were fitted by a non-linear regression using the mathematical model log (EOs) vs. normalized response in GraphPad Prism, (Prism version 6.00 for MS Windows, GraphPad Software, La Jolla California USA, www.graphpad.com). IC_50_ was calculated as the drug concentration required for reducing virus replication by 50%.

### 3.7. Machine Learning Classification Modeling

MATLAB software, Version 9.1.0 (R2016b; The MathWorks, MA, USA), using PCA Toolbox for MATLAB (version 1.3; PCA, unsupervised data analysis) and Classification Toolbox for MATLAB (version 5.0; PLS-DA, supervised data analysis) [35,36] was used for all calculations.

#### 3.7.1. Unsupervised Data Analysis

Chemical composition data was organized in an independent data matrix consisting of 38 rows (EOs samples) and 56 columns (chemical components). PCA [20] was initially applied as a preliminary step for exploratory analysis to identify possible outliers. The number of principal components (PCs) was chosen on the basis of a minimal increment (5%) of explained variance.

#### 3.7.2. Supervised Classification Modeling

For the binary classification models [22], performed with PLS-DA [37], the EOs concentrations, used as the independent variable X matrix, were pretreated by means of a mean scaling. The PLS-DA technique is a special form of projection of latent structures (PLS, also named partial least square) commonly used for linear classification [38] that search for latent variables with a maximum covariance with the Y variables. In PLS-DA the Y-block describes which objects are in the defined classes. In a binary classification application, the continuous variable can be easily defined in two classes by a cutoff value and setting the values to 1 if the objects have Y higher values than the cutoff and 0 if they are lower [39]. Elaboration of the model will return calculated Y values, in a similar way as for a regression approach by PLS. In analogy with the PLS algorithm, the model is described by the variables regression, i.e., for each class. PLS coefficients characterized by high absolute values are generally related to important variables for class discrimination, in particular positive coefficients indicate those variables that most contribute to the increase of the 1 class calculated response [35]. The coefficients were used to elaborate the feature importance plot (see the Results and Discussion Section Figure 7 and Figure 8).

The antiviral activity (IC_50_) and the toxicity (CC_50_; Table 2) values experimentally determined were used as dependent variable vectors in two distinct PLS-DA models in which each dependent variable was divided into two classes (active/non-active and toxic/non-toxic) on the basis of an optimal cut-off value (see results section) obtained by a systematic procedure search. The final classification models were numerically and graphically evaluated through explained variance, accuracy (ACC) and non-error rate (NER) [40] as calculated from the final model and leave-one-out cross internal validation.

The accuracy describes the global predictive ability, identifying as positive the true positive and as negative the true negative and is defined as:

ACC=∑gG=n1gn

where *n* is the total number of samples. Not assigned samples are not considered for the accuracy calculation.

The *NER* [29] was calculated as arithmetic mean of sensitivity values of the *G* classes.

NER=∑gG=S1ngG

where *G* is the total number of classes, and *Sn_g_* [40] is the sensitivity of the *g*-th class, also known as true positive rate, and can be defined as the ability of given classifier to correctly identify the samples of the *g*-th class and can be calculated as:

$$Sn_{g}=\frac{c_{gg}}{n_{g}}$$

where *c_gg_* is the number of samples belonging and correctly assigned to class *g* and *n_g_* is the number of samples belonging to the *g*-th class. In the text a reference was added for this concept.

### 3.8. Assessment of the Models’ Predictive Ability

An internal library of 52 EOs samples not used to define the PLS-DA model was selected as the external test set. The chemical composition of the test set was known and organized in an independent data matrix similarly as for the training set and consisted of 52 rows (EOs samples) and 56 columns (chemical components) [27].

## 4. Conclusions

From an internal library of 90 different EOs a training set of 38 was compiled, tested for antiviral activity and cytotoxicity and the experimental data used to develop PLS-DA classification models able to discriminate either anti-HSV-1 active versus non active samples or cytotoxic versus low cytotoxic endowed samples. Two classification models were obtained with satisfactory statistical coefficients. Analysis of the models by means of features importance indicated β-myrcene, limonene 3-octanol and chrysanthenone as key chemical components for the EOs’ biological effects. The two models were applied to EOs not included in the training set and proved their predictive abilities in selecting five EOs capable of high antiviral potency and low cytotoxicity. Four out of five (80%) of the selected EOs indeed revealed to be active against HSV-1 and with low cytotoxicity values.

These results and those previously reported demonstrating the EOs great antioxidant and antimicrobial properties [41], confirm the possibility of using these substances in a wide array of applications, like pharmaceutical [11], nutraceutical [42] and food preservatives [43]. Despite the wide EOs potential, further efforts are needed to better understand crucial-chemical information like optimum dose and safe limits, as well as aspects related to food uses, as the impact of these compounds on sensory quality.

Different interesting aspects to be clarified and deepened is how the chemical composition may influence the observed biological effects, if these are the results of possible synergistic or antagonistic mechanisms between the single chemical components and if the isolated compound preserves the same identified effects. Several reports with this purpose have been found in the literature, often enriched with extensive machine learning approaches [11] in which a potential main chemical compound was identified and investigated about its biological properties [44]. This latter step is crucial for the detection of new molecules able to replace and support those already known and used as antimicrobial and antifungal agents. In this context are important further extensive researches trying to model blended EOs with enhanced biological profiles and mix key chemical components for preparation of mixture with ad-hoc enhanced efficacy and less toxicity.

## Figures and Tables

**Table 1:** Cross-validation scores for the binary partial least squares discriminant analysis (PLS-DA) classification models built with different IC_50_ cut-off values.

Statistical Parameter	IC_50_-PLS-DA	IC_50_-PLS-DA
cut-off (mg/mL)	0.25	0.20
EV	75%	75%
FNER	0.77	0.68
CVNER	0.71	0.61
ACC	0.76	0.68

EV: explained variance. FNER: fitting-non-error-rate. CVNER: cross-validation-non-error-rate. ACC: accuracy.

**Table 2:** Essential oils’ (EOs’) anti-HSV-1 (IC_50_), cytotoxicity (CC_50_) and selectivity index (SI) of the tested EOs.

# ^a^	EO Id ^b^	IC_50_ (mg/mL)	CC_50_ (mg/mL)	SI	# ^a^	EOs ^b^	IC_50_ (mg/mL)	CC_50_ (mg/mL)	SI
**1**	FA2	0.14	1.45	9.70	**20**	CS6	0.21	2.66	12.48
**2**	FA6	0.16	1.82	11.31	**21**	CO1	0.21	2.50	11.89
**3**	FS1	0.19	1.01	5.17	**22**	CO2	0.33	>3.00	>9.0
**4**	FS2	0.19	0.31	1.61	**23**	CO6	0.17	2.22	12.08
**5**	FS6	0.19	1.51	7.78	**24**	CJM2	0.33	2.50	7.60
**6**	FO1	0.72	1.50	2.07	**25**	CJM5	0.14	>3.00	>22.2
**7**	FO3	0.65	1.02	1.58	**26**	CAM1	0.20	2.65	13.11
**8**	FO6	0.54	1.69	3.14	**27**	CAM3	0.15	2.99	19.50
**9**	FO24	0.58	0.36	0.61	**28**	CAM5	0.44	1.10	2.50
**10**	FOM3	0.18	1.7	9.63	**29**	CSM1	0.17	2.00	11.5
**11**	CJ1	0.27	2.15	7.87	**30**	CSM3	0.24	2.12	8.97
**12**	CJ2	0.14	>3.00	>22.2	**31**	CSM5	0.12	1.93	15.77
**13**	CA1	0.16	2.84	17.54	**32**	COM1	0.28	2.76	9.78
**14**	CA2	0.15	>3.00	>19.8	**33**	COM3	0.31	1.99	6.36
**15**	CA3	0.18	2.50	14.26	**34**	COM5	0.36	2.46	6.74
**16**	CA6	0.14	1.14	7.99	**35**	R6	0.43	0.41	0.94
**17**	CS1	0.14	2.50	17.82	**36**	R24	0.18	0.36	1.99
**18**	CS2	0.21	2.90	14.04	**37**	RM4	0.30	0.90	3.05
**19**	CS3	0.23	2.68	11.61	**38**	RM6	0.20	0.33	1.65

^a^ # EO number. ^b^ EO’s Id refers to that reported in reference [10] published by the same authors of this reports. SI = (CC_50_/IC_50_).

**Table 3:** Cross-validation scores for the binary PLS-DA classification models built with different cut-off values.

	IC_50_-PLS-DA	CC_50_-PLS-DA
cut-off (mg/mL)	0.15	2.06
ONPC	7	3
EV	98%	78%
FNER	0.97	1.00
CVNER	0.85	0.97
ACC	0.87	0.97

ONPC: optimal number of PCs (see text). EV: explained variance. FNER: fitting-non-error-rate. CVNER: cross-validation-non-error-rate. ACC: accuracy.

**Table 4:** Biological activity classes (IC_50_ and CC_50_) of the EOs used as an external test set.

#	EO Id ^a^	IC_50_ Class Predicted	CC_50_ Class Predicted	#	EOs ^a^	IC_50_ Class Predicted	CC_50_ Class Predicted	#	EOs^a^	IC_50_ Class Predicted	CC_50_ Class Predicted
**39**	FA1	NA	T	**57**	FSM5	NA	T	**75**	CJM4	A	NT
**40**	FA3	A	T	**58**	FOM1	A	T	**76**	CAM2	NA	T
**41**	FA12	NA	T	**59**	FOM2	NA	T	**77**	CAM4	NA	T
**42**	FA24	A	T	**60**	FOM4	NA	T	**78**	CSM2	A	T
**43**	FS3	A	T	**61**	FOM5	NA	T	**79**	CSM4	A	NT
**44**	FS12	NA	T	**62**	CJ3	A	T	**80**	COM2	NA	T
**45**	FS24	NA	T	**63**	CJ6	A	T	**81**	COM4	NA	T
**46**	FO2	NA	T	**64**	CJ12	A	T	**82**	R1	NA	T
**47**	FO12	NA	T	**65**	CJ24	A	T	**83**	R2	A	T
**48**	FAM1	A	T	**66**	CA12	A	T	**84**	R3	A	T
**49**	FAM2	NA	T	**67**	CA24	A	T	**85**	R12	NA	T
**50**	FAM3	NA	T	**68**	CS12	A	T	**86**	R30	NA	T
**51**	FAM4	NA	T	**69**	CS24	A	T	**87**	RM1	NA	T
**52**	FAM5	NA	T	**70**	CO3	NA	T	**88**	RM2	NA	T
**53**	FSM1	NA	T	**71**	CO12	NA	T	**89**	RM3	NA	T
**54**	FSM2	A	T	**72**	CO24	A	T	**90**	RM5	NA	T
**55**	FSM3	NA	T	**73**	CJM1	A	NT				
**56**	FSM4	NA	T	**74**	CJM3	A	NT				

^a^ EO’s Id refers to that reported in reference [10].

**Table 5:** Experimental results obtained from biological assays on the predicted samples (see text).

#	EO Id ^a^	IC_50_ (mg/mL)	CC_50_ (mg/mL)	SI
**68**	CS12h	0.460	0.520	1.1
**73**	CJM1	0.063	>3	>47.5
**74**	CJM3	0.143	2.503	17.5
**75**	CJM4	0.116	>3	>25.9
**79**	CSM4	0.124	>3	>24.2

^a^ EO’s Id refers to that reported in reference [10].
